# Community pharmacists workforce readiness to deliver vaccination services: A cross‐sectional study from Jordan

**DOI:** 10.1002/prp2.943

**Published:** 2022-03-03

**Authors:** Saja A. Alnahar, Georgios Gkountouras, Rula M. Darwish, Ian Bates

**Affiliations:** ^1^ Department of Clinical Pharmacy and Pharmacy Practice Faculty of Pharmacy Yarmouk University Irbid Jordan; ^2^ Department of Primary Care and Public Health Faculty of Medicine Imperial College London London UK; ^3^ Division of Population Health Health Services Research and Primary Care School of Health Sciences The University of Manchester Manchester UK; ^4^ Department of Pharmaceutics and Pharmaceutical Technology School of Pharmacy University of Jordan Amman Jordan; ^5^ School of Pharmacy University College London London UK

**Keywords:** Community pharmacies, Jordan, pharmacists readiness, pharmacist‐vaccinators, workforce

## Abstract

This study assesses Jordanian community pharmacists’ readiness and willingness to deliver vaccination services in their practice sites. Between February and April 2021, a self‐administered online questionnaire was distributed via social media, WhatsApp messages, and personal communication. The questionnaire targeted practicing community pharmacies. Descriptive and inferential data analysis was carried out. A total of 403 community pharmacists participated in the study. Almost 146 (36%) community pharmacists reported vaccinating patients in their practice sites. However, readiness assessment revealed that only 54 (13.4%) pharmacists received the required training and qualifications. Moreover, 33 (8.2%) study participants worked in adequately equipped and designed community pharmacies. Overall, surveyed participants held positive attitudes toward their involvement in vaccination services: 260 (64.5%) pharmacists were willing to vaccinate patients, and 227 (65.0%) out of unready, unqualified, participants were willing to get needed training and qualifications. According to study participants, regulatory and professional bodies (Ministry of Health, Jordan Pharmacists Association) are influential in supporting pharmacist‐vaccinators. Among the investigated factors, organizational structure and employment status were significantly associated with pharmacists’ readiness to deliver vaccination. This study revealed that further work is needed to increase pharmacists’ and pharmacies’ readiness to deliver vaccination services and that regulators should follow a more active approach in highlighting the importance of training and the impact of training in patients’ safety and satisfaction.

## Graphical abstract

This figure briefly describes the study's population, design, and main outcomes.

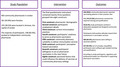

**Abbreviations**
CI: confidence interval
CPR: cardiopulmonary resuscitation
JPA: Jordan Pharmacists Association
MoH: Ministry of Health
N: number
SD: standard deviation
WHO: World Health Organization

## 1 INTRODUCTION

Seasonal influenza is a highly infectious viral disease that affects different age groups and demographics, including the elderly, paediatrics, pregnant women, and healthcare workers.^1^, ^2^, ^3^, ^4^ In 2017, the World Health Organization (WHO) reported that up to 650 000 people died due to complications associated with respiratory diseases from seasonal influenza.^5^ Seasonal influenza is a resource‐demanding disease that can burden national healthcare systems. The study of Ozawa et al estimated that seasonal influenza costs the United States healthcare system $5.8 billion annually.^6^ Furthermore, in Germany, the direct cost of seasonal influenza was estimated to be more than €78 million.^7^

Seasonal influenza is classified as a preventable communicable disease, which can be avoided through vaccination and adhering to proper respiratory hygiene practices.^8^, ^9^ Vaccination is an effective tool in reducing the number of flu cases, leading to lower number of hospitalizations and lower overall cost of the seasonal flu.^10^, ^11^ There is no reported prevalence or incident rate of seasonal influenza in Jordan. However, In 2017, the prevalence rate of the upper respiratory tract, including seasonal influenza, was 2.52%.^12^ The Ministry of Health (MoH) actively monitors seasonal flu cases in Jordan.^13^ In addition to prevalence and incident rate monitoring, the MoH has published a series of educational and informative materials that included explaining seasonal influenza signs and symptoms, explaining precautionary measures to avoid seasonal influenza, and emphasizing the importance of vaccination.^14^

Until July 2020, only clinics, hospitals, and primary healthcare centers were eligible to deliver vaccination services. As a result, pharmacists’ role was limited to advocacy for vaccination and patient counseling.^15^ However, on August 17, 2020, the MoH allowed pharmacists to vaccinate patients against seasonal influenza at community pharmacy settings. This decision was considered an expansion of community pharmacists’ roles and responsibilities, and it was the result of the Jordanian Pharmacists Association (JPA) efforts to promote pharmacists as pharmacist‐vaccinators.^16^

Jordan adopted the practice of many other countries which have listed pharmacists as pharmacist‐vaccinators, such as the United Kingdom, Australia, Switzerland, and Portugal.^15^, ^17^, ^18^ Pharmacists’ involvement in vaccination programs provided more accessible and convenient alternatives than physicians’ clinics.^17^ Moreover, administering vaccines at community pharmacies led to an increase in the uptake of vaccines.^18^, ^19^

In Jordan, the BPharm and the PharmD academic curricula include pharmacy practice experience (PPE) courses, where students are expected to shadow, observe and be trained by practicing experienced pharmacists in their practice sites, whether community pharmacies or hospital wards. During the PPE courses, students learn and are trained on how to dispense prescriptions, carry out patients’ counseling, detect any medicines‐related problems and review therapeutic plans. However, the PPE courses do not include training on vaccination administration. Moreover, the majority of pharmacy faculties do not offer training on vaccination as part of their academic programs.

The Jordanian MoH mandated that only sufficiently qualified and adequately trained pharmacists are allowed to administer influenza vaccines. The MoH has also specified the criteria needed to consider a community pharmacy as a suitable vaccine administration site. Therefore, the JPA has published its immunization guidelines, which details the training requirements for pharmacist‐vaccinators, and the equipment and tools needed to be within a community pharmacy to be considered as a suitable vaccination site.^16^

As per the JPA guidelines, for a pharmacist to be sufficiently qualified, he/she needs to be trained on vaccination administration, first aid procedures and cardiopulmonary resuscitation (CPR) performance and have read the JPA guidelines. Moreover, a pharmacist‐vaccinator needs to practice immunization in a suitably equipped community pharmacy, which has vaccination specific space, a refrigerator specific for vaccines, temperature monitor, portable refrigerator in case of power failure, anaphylaxis response kit, anaphylaxis management poster, safety box, medical waste bin, materials for hand sanitization and surface cleaning and vaccinated patients record. Pharmacists, who want to be qualified pharmacist‐vaccinators, should get training courses offered by the JPA or any other accredited training agencies.

## 2 STUDY AIM AND OBJECTIVES

This study aims to assess Jordanian community pharmacists’ current vaccination practices, their readiness to act as pharmacist–vaccinators, and their pharmacies’ suitability for vaccination services. In addition, the study aims to identify pharmacists’ perception of vaccination delivery at community pharmacies and perceived enablers and barriers.

## 3 METHODS

### 3.1 Study design

This study is a cross‐sectional study of licensed and practicing community pharmacists in Jordan. Eligible participants were identified using the Jordan Pharmacists Association (JPA) database and personal connections.

### 3.2 Study settings and participants

All practicing community pharmacists in Jordan were eligible for this study. According to the JPA 2019 database, 7525 pharmacists work in community pharmacy settings. Therefore, a minimal sample of 366 pharmacists is needed for this study. The sample size was calculated based on a 50% expected frequency and a 5% confidence limit. This minimal sample would give adequate power for bivariate, multivariable analysis to be carried out.

### 3.3 Survey design

Data collection was carried out using a questionnaire instrument developed based on an extensive literature review, research aim and objectives, and the JPA community pharmacists’ immunization guidelines. The initial draft went through content and face validity assessment exercise. The validity check was carried out over two stages. Stage one: the questionnaire was reviewed by a panel of eight experts; the panel included five academics specializing in pharmacy practice or clinical pharmacy and three practicing community pharmacists who were involved in developing the JPA immunization guidelines. The questionnaire instrument was revised and updated based on reviewers’ comments and advice. Stage two: the revised instrument was piloted on a convenience sample of 17 pharmacists; volunteers were asked to assess the instrument's comprehensiveness, readability, and follow. After piloting, further amendments were considered. Pilot data were not included in the final analysis.

The final questionnaire instrument contained 23 questions grouped into eight constructs as per the following: first construct: pharmacists’ demographic data such as age and gender. Second construct: participants’ employment details such as type of community pharmacy (independent vs chain pharmacy), number of working hours, and years of experience. Third construct: current immunization practices such as vaccines administered, targeted age group, and requested fees. Fourth construct: participants’ readiness to serve as pharmacist‐vaccinators in terms of received training and qualifications. Fifth construct: related to the practice site, community pharmacy, suitability to deliver immunization services in terms of available equipment and facilities. Sixth construct: participants’ perception toward vaccination. Seventh construct: participants’ willingness to act as pharmacist‐vaccinators in the near future. Eighth construct: perceived factors that could influence the delivery of vaccines at a community pharmacy setting.

### 3.4 Survey distribution and administration

The final survey instrument was self‐administered using the Qualtrics XM^®^ platform (Qualtrics, 2020).^20^ In the period between February and April 2021, the targeted eligible participants were sent the survey link via email, WhatsApp messages, and text messages.

Different approaches and strategies were deployed to identify and recruit eligible participants; these strategies were: (i) approaching community pharmacists directly by the research team, (ii) posting survey link on social media pages related to the JPA and other Jordanian pharmacists groups, and (iii) contacting community pharmacists through the chairs of JPA regional committees and sub‐committees on behalf of the research team.

### 3.5 Statistical analysis

Following data collection, data were extracted and logged in an Excel^®^ workbook (Microsoft Office MS, 2013). Before analysis, data cleaning, coding, and grouping were carried out.

A pharmacist's readiness to act as a pharmacist‐vaccinator was determined based on the pharmacist's qualifications and certifications. For example, if a pharmacist had received all required training, as per the JPA guidelines, and had read the guidelines, the pharmacist was considered a qualified pharmacist‐vaccinator, ready to vaccinate. On the other hand, a community pharmacy was considered suitable to deliver vaccination services if all required equipment and facilities were reported to be available. Finally, pharmacists’ willingness to be qualified pharmacist‐vaccinators was determined by their preparedness to receive needed training and certificates.

Participants’ perceptions toward delivering vaccination services by community pharmacists at a community pharmacy settings were assessed using a five‐point Likert scale. However, to facilitate data analysis, the scale was converted into a three‐point scale. Therefore, the first two categories (strongly agree and agree) were grouped into one (agree), the last two categories (strongly disagree and disagree) were grouped into one (disagree), the intermediate scale (neither) was left as it is.

Data were analyzed descriptively using frequencies, percentages, and standard deviation, when applicable. Additionally, inferential analysis and logistic regression were used to determine factors associated with pharmacists’ readiness to act as pharmacist‐vaccinators and community pharmacies suitability for immunization services. Data analysis was carried out using STATA^®^ data analysis and statistical software (StataCorp, 2016).

### 3.6 Ethical consideration

The Research Ethics Committee at Jordan University of Science and Technology (JUST) and King Abdulla University Hospital (KAUH), Irbid, Jordan, reviewed and approved this study (Reference No.: 17/135/2020).

## 4 RESULTS

### 4.1 Participants demographic and characteristics

In total, 403 community pharmacists participated in this study. However, as the distribution of this study was based on different approaches to identify and recruit eligible participants, including social media, it was not possible to calculate the response rate.

Out of 403 participants, 261 (64.8%) were females, and almost 40% were located in Amman, the capital of Jordan. The majority of participants, 338 (83.9%), worked at independent community pharmacies, and 256 (64.3%) were employees. In addition, 278 (68.9%) worked more than 36 h weekly, and almost 25% handled more than 20 prescriptions per day. Table 1 summarizes participants’ demographics and characteristics.

**TABLE 1:** Participants’ characteristics and demographics

Investigated attributes	*N* (%)
Number of participants	403 (100%)
Gender	
Females	261 (64.8%)
Males	133 (33%)
Prefer not to say	9 (2.2%)
Participants’ age (years), mean ± SD	33.93 ± 10.81
Degree of qualification	
BPharm	375 (93.1%)
PharmD	28 (6.9%)
Years of experience	
Less than 1 year	49 (12.2%)
1–5 years	156 (38.7%)
6–10 years	49 (12.2%)
11–15 years	31 (7.7%)
16–20 years	33 (8.2%)
21–25 years	43 (10.7%)
26–30 years	27 (6.7%)
More than 30 years	15 (3.7%)
Employment status	
Owner	144 (35.7%)
Employee	259 (64.3%)
Type of practice site	
Independent community pharmacy	338 (83.9%)
Chain community pharmacy	65 (16.1%)
Location (Governorate)	
Amman	155 (38.5%)
Ajloun	12 (3%)
Aqaba	11 (2.7%)
Balqa	16 (4%)
Irbid	103 (25.6%)
Jerash	11 (2.7%)
Karak	13 (3.2%)
Ma'an	9 (2.2%)
Madaba	13 (3.2%)
Mafraq	18 (4.5%)
Tafilah	9 (2.2%)
Zarqa	33 (8.2%)
Type of area	
City	329 (81.6%)
Village	62 (15.4%)
Badia	12 (3%)
Weekly working hours	
Less than 24 h	38 (9.4%)
From 24–36 h	87 (21.6%)
From 36–48 h	169 (41.9%)
More than 48 h	109 (27.0%)
Number of handled prescriptions per day	
Less than 10 prescriptions	138 (34.2%)
From 10–19 prescriptions	161 (40%)
From 20–30 prescriptions	56 (13.9%)
More than 30 prescriptions	48 (11.9%)
Total number of employees at the practice site*	
1	50 (12.4%)
2	138 (34.2%)
3	129 (32.0%)
4	44 (10.9%)
5–10	38 (9.4%)
More than 10	4 (1.0%)

Abbreviations: *N*, number; SD, standard deviation.

* Including the participant.

### 4.2 Current vaccination practice

**FIGURE 1:**
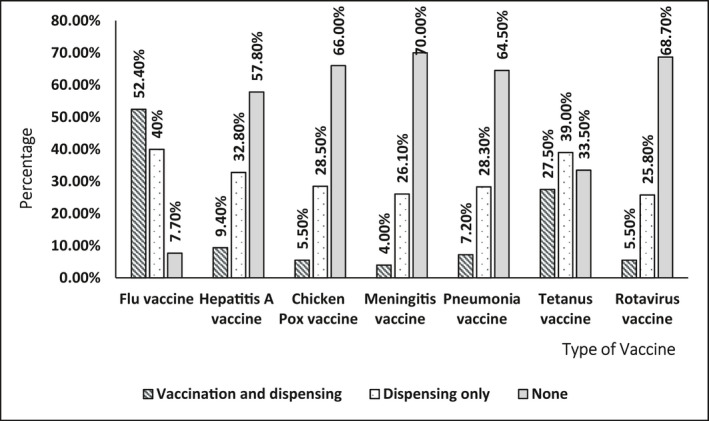
Vaccines administered in community pharmacy settings

Out of the 403 participants, only 146 (36.2%) reported that they were, in general, practicing vaccination (pharmacist‐vaccinators) in their community pharmacies. Results showed that the majority, 137 (93.8%), of pharmacist‐vaccinators were vaccinating adults only, while 30% of vaccinating pharmacists asked for vaccination fees. The flu vaccine was the most commonly administered, while meningitis vaccines were the lowest (Figure 1 **)**. When it comes to dispensing, the rotavirus vaccine was the least frequently dispensed vaccine. Table 2 summarizes vaccination practices by community pharmacists in Jordan.

**TABLE 2:** Vaccination practices by community pharmacists in Jordan

Investigated attributes	*N* (%)
General vaccination practice	
Practicing vaccination	146 (36.2%)
Not practicing vaccination	257 (63.8%)
Seasonality of vaccination	
Seasonally	115 (78.7%)
Year long	31 (21.2%)
Vaccination fee	
Extra fee added	44 (30.1%)
No extra fee added	102 (69.9%)
Vaccination requested by	
Patient request	123 (84.2%)
Physician request	23 (15.8%)
Targeted age group	
Younger than 2 years old	7 (4.8%)
From 2 to 17 years old	2 (1.4%)
From 18 to 65 years old	132 (90.4%)
Older than 65 years old	5 (3.4%)

### 4.3 Pharmacists readiness and community pharmacies suitability to deliver vaccination services

Assessing pharmacists’ readiness was based on received training courses as reported by the participants themselves. Study findings revealed that 202 (50.1%) received training on vaccination, and 177 (43.9%) participants had read the JPA immunization guidelines. However, the overall readiness assessment showed that only 54 (13.4%) participants were qualified enough to be pharmacist‐vaccinators; these pharmacists received training on vaccination, CPR performing, and first aid procedures, and had read the JPA guidelines. Furthermore, evidence showed that only 30 (20.5%) out of the 146 pharmacist‐vaccinators were sufficiently qualified and trained.

Assessing community pharmacies suitability for vaccination delivery showed that the majority of participants worked in community pharmacies which had vaccination‐specific place/room, a refrigerator specific for vaccines and temperature monitor. However, only quarter of participants were working at community pharmacies which had anaphylaxis response kits. Interestingly, out of the 146 vaccination practicing pharmacists, only 12 (8.2%) worked at immunization‐suitable community pharmacies. Additionally, only 8 (5.5%) of the research participants were sufficiently qualified and worked in suitably equipped community pharmacies. Table 3 summarizes participating pharmacists’ readiness and suitability of their pharmacies.

**TABLE 3:** Participating pharmacists’ readiness and suitability of their pharmacies

Investigated attributes	Yes *N* (%)	No *N* (%)
Pharmacists readiness		
First aid training	164 (40.7%)	239 (59.3%)
CPR training	125 (31.0%)	278 (69.0%)
Immunizing qualification training	202 (50.1%)	201 (49.9%)
Read the JPA vaccination manual	177 (43.9%)	226 (56.1%)
Pharmacies suitability		
Vaccination specific place/room	319 (79.2%)	84 (20.8%)
Refrigerator specific for vaccines	368 (91.3%)	35 (8.7%)
Temperature monitor	346 (85.9%)	57 (14.1%)
Portable refrigerator in case of power failure	284 (70.5%)	119 (29.5%)
Anaphylaxis response kit	107 (26.6%)	296 (73.4%)
Anaphylaxis management poster/guidance	82 (20.3%)	321 (79.7%)
Safety box	205 (50.9%)	198 (49.1%)
Medical waste bin	238 (59.1%)	165 (40.9%)
Materials for hand sanitisation and surface cleaning	384 (95.3%)	19 (4.7%)
Vaccinated patients record	83 (20.6%)	320 (79.4%)

### 4.4 *Pharmacists willingness to be pharmacist*‐*vaccinators*

In addition to assessing pharmacists and pharmacies’ readiness to deliver vaccination services, the study investigated pharmacists’ willingness to deliver vaccinating services and get the necessary qualification and equipment needed to vaccinate patients in community pharmacy settings. Results showed that 260 (64.5%) pharmacists were willing to vaccinate patients. In addition, 139 (54.1%) participants out of the 257 nonvaccinating pharmacists showed an interest in practicing vaccination in the near future. Moreover, 170 (66.1%) participants of the nonvaccinating pharmacists stated that they would encourage patients to get vaccinated in community pharmacy settings.

Interestingly, 227 (65.0%) out of unready, unqualified, participants were willing to get the required training and qualifications. In terms of suitability of pharmacy premises, 207 (69.9%) of the 269 participants, who did not have an anaphylactic shock kit, were planning to get the kit. In addition, emerging data showed that 40 (47.6%) of pharmacists, who did not have vaccination‐specific room/space, reported that they would have a dedicated space for vaccination to maintain patients’ privacy. Finally, 38 (37.3%) of participants, who were not asking for a vaccination fee, showed an interest in having additional fees for vaccination. Table 4 summarizes participating pharmacists’ future intentions with regards to vaccination services.

**TABLE 4:** Participants future intentions toward vaccination services

Investigated attributes	Highly likely *N* (%)	Likely *N* (%)	Do not know *N* (%)	Unlikely *N* (%)	Highly unlikely *N* (%)
Will practice vaccination in the future	108 (26.8%)	152 (37.7%)	65 (16.1%)	39 (9.7%)	39 (9.7%)
Will encourage patients to get vaccinated	138 (34.2%)	158 (39.2%)	49 (12.2%)	30 (7.4%)	28 (6.9%)
Will get first aid training	171 (42.4%)	142 (35.2%)	43 (10.7%)	20 (5%)	27 (6.7%)
Will get training related to vaccination	167 (41.4%)	151 (37.5%)	36 (8.9%)	24 (6%)	25 (6.2%)
Will get CRP training	157 (39%)	145 (36%)	48 (11.9%)	24 (6%)	29 (7.2%)
Will get an anaphylactic shock kit	139 (34.5%)	148 (36.7%)	55 (13.6%)	28 (6.9%)	33 (8.2%)
Will ask for a fee	75 (18.6%)	112 (27.8%)	94 (23.3%)	75 (18.6%)	47 (11.7%)
Will have a private place	169 (41.9%)	142 (35.2%)	38 (9.4%)	23 (5.7%)	31 (7.7%)
Will have an appointment system	107 (26.6%)	157 (39%)	55 (13.6%)	44 (10.9%)	40 (9.9%)

### 4.5 Pharmacists perception toward vaccination services

Assessing participants’ perception toward pharmacy‐based immunization revealed that more than 50% of the research participants showed a positive attitude toward pharmacists’ involvement in immunizing activities. Data showed that there was a significant difference between pharmacist‐vaccinators and nonvaccinators, while 113 (77.4%) of pharmacist‐vaccinators believed that community pharmacists should be practicing immunization in their pharmacies, only 109 (42.4%) of nonvaccinating pharmacists held the same belief. Vaccination fees were not highly demanded by the study participants, as only 45 (44.1%) of pharmacist‐vaccinators, who did not ask for vaccination fees, believed pharmacists should be paid for their vaccination services.

In general, the majority of the research participants, more than 70% of the respondents, thought that patients positively perceived pharmacists’ involvement in vaccination services in terms of being trusted as pharmacist‐vaccinators and saving patients’ time to getting vaccinated.

Concerning training and qualifications, 288 (82.5%) of unready pharmacists believed that pharmacists should be adequately trained to deliver vaccination services. Moreover, 299 (80.8%) of participants working in unsuitably equipped pharmacies thought that community pharmacists should be sufficiently equipped to deliver vaccination services to the patients. Table 5 summarizes participants’ perceptions toward vaccination service in community pharmacy settings.

**TABLE 5:** Participants’ perceptions toward vaccination services and influencing factors

Participants’ Perceptions	Strongly agree *N* (%)	Agree *N* (%)	Neutral *N* (%)	Disagree *N* (%)	Strongly disagree *N* (%)
Pharmacists should vaccinate patients	81 (20.1%)	141 (35%)	88 (21.8%)	51 (12.7%)	42 (10.4%)
Pharmacy‐based vaccination could be dangerous	19 (4.7%)	107 (26.6%)	122 (30.3%)	112 (27.8%)	43 (10.7%)
Pharmacists should be paid	55 (13.6%)	148 (36.7%)	108 (26.8%)	61 (15.1%)	31 (7.7%)
Patients prefer to get their vaccines at the pharmacy to save time	104 (25.8%)	189 (46.9%)	56 (13.9%)	32 (7.9%)	22 (5.5%)
Patients trust the pharmacist to vaccinate them	90 (22.3%)	206 (51.1%)	73 (18.1%)	22 (5.5%)	12 (3%)
Pharmacists should be trained on how to vaccinate patients	171 (42.4%)	161 (40%)	38 (9.4%)	16 (4%)	17 (4.2%)
Pharmacists should encourage patients to get vaccinated	151 (37.5%)	195 (48.4%)	39 (9.7%)	5 (1.2%)	13 (3.2%)
Pharmacists should be fully equipped	147 (36.5%)	176 (43.7%)	49 (12.2%)	14 (3.5%)	17 (4.2%)

**FIGURE 2:**
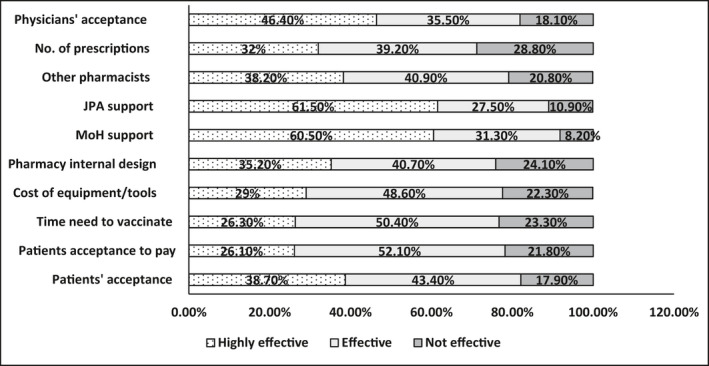
Participants’ perceptions toward influencing factors

Investigating factors that might influence the delivery of vaccination services showed that all listed factors were considered influential by the vast majority of the research participants. Figure 2 summarizes participants’ perceptions toward influencing factors.

### 4.6 Predictors of pharmacists’ and pharmacies readiness

Regression analysis showed that the type of area (rural vs urban), type of pharmacy (independent vs chain), or the number of pharmacists had no significant association with community pharmacy suitability to deliver vaccination services. On the other hand, pharmacists’ readiness was significantly associated with the number of pharmacists working at the pharmacy and the participant's working status (employer vs owner). While being an owner was negatively associated with a pharmacist's readiness to vaccinate (Coefficient = −1.054, 95% confidence interval [CI]: −2.046 to 0.062), having more co‐workers increased the likelihood of being sufficiently trained and qualified (Coefficient = 0.174, 95% confidence interval [CI]: 0.026–0.321). Tables 6 and 7 summarize regression analyses output.

**TABLE 6:** Predictors of community pharmacy readiness as a vaccination site

No. of observations (*N* = 403)			
Variables	Coefficient	*p*‐value	95% CI
Pharmacy type			
Chain pharmacy	Reference category		
Independent pharmacy	0.797	.254	(−0.571 to −2.165)
Area			
Rural	Reference category		
Urban	0.792	.209	(−0.444 to −2.028)
No. of pharmacists	0.075	.298	(−0.066 to −0.217)
Constant	−4.028	.000	(−5.648 to −2.406)

Abbreviations: CI, confidence interval; *N*, number.

**TABLE 7:** Predictors of community pharmacists’ readiness to act as pharmacist‐vaccinators

No. of observations (*N* = 394)			
Variables	Coefficient	*p*‐value	95% CI
Gender			
Male	Reference category		
Female	0.056	.878	(−0.663 to 0.776)
Years of experience	−0.013	.604	(−0.061 to 0.035)
Pharmacy type			
Chain pharmacy	Reference category		
Independent pharmacy	0.692	.263	(−0.519 to 1.903)
Area			
Rural	Reference category		
Urban	0.261	.589	(−0.686 to 1.209)
Prescription number			
From 10 to 19 prescriptions	Reference category		
Less than 10 prescriptions	−0.685	.075	(−1.439 to 0.070)
From 20 to 30 prescriptions	−0.046	.921	(−0.948 to 0.856)
More than 30 prescriptions	0.130	.779	(−0.776 to 1.036)
Working hours			
From 36 to 48 h	Reference category		
From 24 to 36 h	0.783	.051	(−0.003 to 1.568)
Less than 24 h	−0.305	.623	(−1.521 to 0.911)
More than 48 h	0.730	.073	(−0.069 to 1.528)
Number of pharmacists	0.174	.021*	(0.026 to 0.321)
Employment status			
Owner	Reference category		
Employee	−1.054	.037*	(−2.046 to −0.062)
Constant	−2.761	.007	(−4.775 to −0.746)

Abbreviations: CI, confidence interval; *N*, number.

* *p* < .05.

## 5 DISCUSSION

By having different roles in the vaccination services, advocates, facilitators, or administrators, pharmacists play a central role in increasing the vaccination rate and protecting the general population. To the best of the research team's knowledge, this is the first study to assess Jordanian community pharmacists’ readiness and willingness to deliver vaccination services in community pharmacies. The study was able to get feedback from 403 practicing community pharmacists using an online self‐administrative questionnaire instrument. The instrument captured data related to general vaccination practices by community pharmacists, community pharmacists’ readiness and willingness to act as pharmacist‐vaccinators, suitability of community pharmacies to deliver vaccination services, and factors that might influence the delivery of vaccination in community pharmacy settings.

Emerging evidence showed that pharmacist‐vaccinators were mainly involved in administering flu vaccines and delivered their services to adult patients regarding the current vaccination practice. This could be attributed to the fact that the national immunization program targets pediatric patients, and it does not include flu vaccine as a mandatory vaccine to all age groups.^21^ Similar to Hastings et al study,^22^ results showed that vaccination was practiced only by one‐third of the study participants. The relatively limited involvement of pharmacists in immunization services can be explained that pharmacists were recently officially authorized to vaccinate patients in community pharmacy settings in Jordan. Furthermore, the national immunization programs led by the MoH and are mainly carried out in primary healthcare centers.^21^ Moreover, the flu vaccine's most frequently administered vaccine has a coverage rate that ranges from 9.9% to 27.5% of the general population.^23^

**FIGURE 3:**
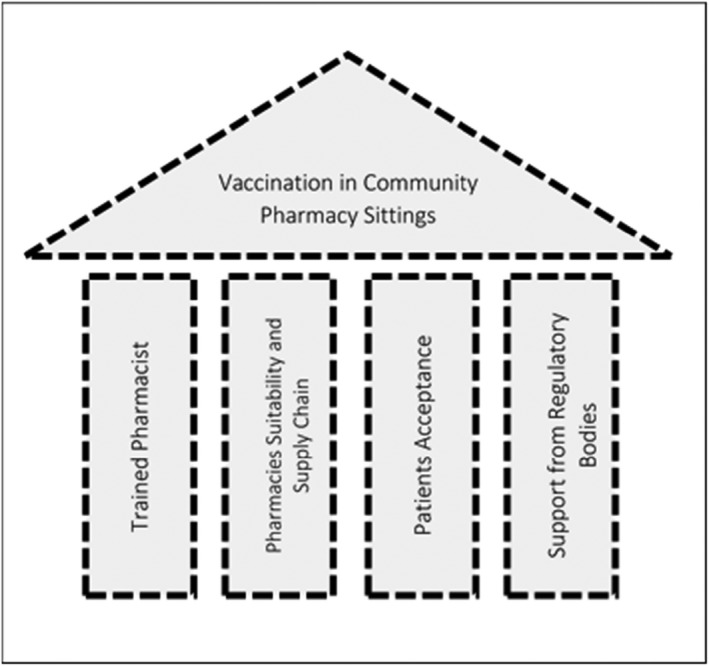
Pillars of vaccination service in community pharmacy settings

The delivery of vaccination services in community pharmacies is based on four pillars: trained and qualified pharmacists, pharmacies and supply chain suitability, patients’ acceptance, and support from regulatory bodies.^24^, ^25^, ^26^, ^27^, ^28^, ^29^, ^30^ Each of these pillars influences each other and is necessary to ensure safe and proper delivery of vaccination services in community pharmacies. Figure 3 summarizes pillars of vaccination services in community pharmacies.

Training and upskilling pharmacists are necessary to enable pharmacists to administer vaccines, enhance pharmacists’ knowledge of the vaccines, and augment their confidence to vaccinate patients.^25^, ^31^ Additionally, available evidence indicates that qualified and trained pharmacists have a higher tendency and willingness to administer vaccines in their pharmacies.^32^ In this study, half of the participants reported that they had received training on administering vaccines. However, only 13.4% received all required training to be qualified pharmacist‐vaccinators. The lack of training and proper qualifications could limit pharmacists’ ability to deliver vaccination services and subject them to legal and ethical liabilities. Additionally, in their study, Batarseh et al showed that the Jordanian population would only allow sufficiently qualified pharmacists to vaccinate them and their children.^28^ Therefore, policymakers and professional leaders need to maximize their efforts in ensuring that practicing community pharmacists are appropriately qualified and that the pharmacists have access to training sessions and workshops.

Pharmacists can play several roles in delivering vaccines; pharmacists can be advocates (educators), facilitators or administrators.^24^, ^33^, ^34^ In this study, the majority of the participants were willing to play the dual role of advocates and administrators. Results showed that, similar to the global trend, community pharmacists are interested and willing to expand their role and be more involved in direct‐to‐patient services and care.^30^, ^35^, ^36^ Additionally, as the majority of unqualified participants were willing to get necessary training and qualifications, this could increase the percentage of pharmacist‐vaccinators in Jordan, improve patients’ accessibility to vaccination services, and increase the vaccination coverage rate.

In addition to assessing pharmacists’ readiness and willingness to incorporate vaccination services in their day‐to‐day, it is of value to assess community pharmacists’ perceptions and attitudes toward vaccination service. Pharmacists’ perceptions and attitudes could influence their decision to deliver the service in their practice site.^37^ Results showed that more than half of the study participants had a generally positive attitude toward vaccination service and the needed qualifications, equipment, and facilities. A welcoming attitude could lead to the actual implementation and delivery of a service.^38^

A sufficiently trained and qualified pharmacist is not enough to ensure vaccination service's safe and proper delivery. Pharmacist‐vaccinators need to operate in practice settings that are adequately equipped and supplied to ensure the safety and satisfaction of patients. The frequently reported needed facilities and equipment is designated room for vaccination, vaccination‐related supplies, and cold‐chain equipment.^24^, ^25^, ^29^, ^32^ Results showed that the majority (80%) of the research participants were working in community pharmacies that have a vaccination‐specific room/place. Moreover, more than half of the study population (63%) had all needed cold‐supply chain devices and equipment in terms of refrigerators specific for vaccines, temperature monitors, and portable refrigerators in case of power failure. However, although having an anaphylactic shock kit is widely recommended by global and national guidelines,^16^, ^39^ only 33% of pharmacist‐vaccinators had the kits in their pharmacies. The absence of an anaphylactic shock kit could jeopardize patients’ safety and subject the pharmacists to legal and ethical liabilities and responsibilities. Finally, results showed that only a minority of the study participants practiced in fully equipped community pharmacies. The results confirmed the general population's perception that most community pharmacies in Jordan do not have the facilities needed to administer vaccines.^28^

The third pillar of the vaccination services in community pharmacy settings is the patients’ acceptance and perception toward pharmacist‐vaccinators. Patients’ acceptance is critical in implementing pharmacy‐based vaccination. Study participants believed that Jordanian patients trust them to deliver vaccination services and that pharmacy‐based vaccination is more convenient for the patients and could save their time. Moreover, half of the participants believed that pharmacist‐vaccinators should be paid and reimbursed for their services. Results aligned with Batarseh et al findings, who reported that more than 70% of Jordanians are willing to pay for vaccination services delivered to them or their children by trained pharmacists.^28^

The last pillar of pharmacy‐based vaccination is the regulatory bodies and agencies. Having a legislative and regulatory framework could provide the needed support for pharmacists to deliver vaccination services in community pharmacy settings.^24^, ^29^ In addition, an official legal and regulatory framework would define the role and responsibilities of pharmacist‐vaccinators and their scope of activity. Based on the previous studies carried out in Jordan, the Jordan Pharmacists Association (JPA) and the MoH were considered to be influential in implementing pharmacy‐based vaccination services.^26^, ^27^ Similarly, in this study, the majority of the study participants considered the support provided by the JPA and the MoH to be influential or highly influential.

Finally, available evidence showed that pharmacists’ readiness to deliver vaccination services was significantly related to organizational structure‐related factors, namely employment status and a number of co‐workers. The study findings are confirmed by Burson et al systematic review, who found that managerial and structure support could increase the feasibility of vaccination delivery and improve vaccination rate in community pharmacy settings.^29^

The impact of pharmacists’ involvement in immunization services is manifested in three main dimensions; the patients, the healthcare system, and the pharmacy profitability.^24^, ^29^, ^34^ Firstly, available evidence shows that pharmacist‐vaccinators could increase the vaccination rate among the general population, especially among elderly patients. Secondly, pharmacists could ease the healthcare systems’ burden and provide a cost‐effective approach to vaccination delivery. Lastly, pharmacist‐vaccinators could increase the profitability of their community pharmacies and their market survival.^24^, ^29^, ^33^, ^34^, ^40^, ^41^

Recently, for its nationwide COVID19 vaccination campaign, the MoH relied on healthcare professionals and medical student volunteers, including pharmacists and pharmacy students, to deliver COVID19 vaccines. These volunteers got sufficient training offered by the MoH and delivered vaccines under medical supervision at the MoH COVID19 vaccination stations. Trained pharmacists and pharmacy students were instrumental in delivering COVID19 vaccines. The MoH offered the training for free, and several training sessions were carried out during the last year. However, as pharmacists and pharmacy students were only trained on vaccinating patients, it is still unclear if they will get training on CPR and first‐aid procedures. Moreover, there is a need to investigate factors influencing pharmacists’ willingness to deliver immunization services in their community pharmacies, such as the cost of training courses and the time needed to complete needed training.

This study assessed the readiness and willingness of Jordanian community pharmacists to deliver vaccination services in their pharmacies. The overall assessment showed that work needs to be done to increase pharmacists and pharmacies readiness to deliver the vaccination services. For example, regulators should follow a more active approach in highlighting the importance of training and how it could impact patients’ safety and satisfaction. Additionally, regulators might need to inspect pharmacist‐vaccinators practice sites to ensure that needed supplies and equipment are available.

## 6 LIMITATIONS

As this study was based on a self‐administered questionnaire, there are a number of inherited design‐related limitations. These limitations include the absence of open‐ended questions, where participants could add aspects related to their experiences in delivering the vaccination services or discuss factors that could enable or hinder the service delivery.

Although this study captured input from community pharmacists working in all governorates in Jordan, the majority of the study participants were located in Amman or Irbid. This did not allow for a meaningful comparison between governorates or between urban and rural locations.

Further research is needed to investigate the approaches and interventions needed to increase the percentage of adequately qualified pharmacists and the percentage of well‐equipped community pharmacies.

## 7 CONCLUSION

The implementation and delivery of vaccination services in community pharmacy settings is influenced by the pharmacists, the pharmacy design and supplies, patients’ acceptance, and legal and legislative framework. Having appropriately trained and sufficiently qualified pharmacists operating in adequately supplied and designed community pharmacies could ensure patients’ safety and satisfaction. Additionally, pharmacy structure‐related aspects could facilitate or hinder the delivery of vaccination services. Lastly, the support provided by regulatory bodies and agencies could assure safe and effective delivery of vaccination services.

This study showed that, in general, Jordanian community pharmacists have a positive attitude toward pharmacy‐based vaccination services. Moreover, a significant proportion of the study participants were willing to get the needed training and qualifications and equip their practice site with necessary supplies and equipment.

The study can be enhanced by looking into factors hindering the adoption and implementation of vaccination services in Jordanian community pharmacy settings. It would be of value to look into the motives of ready and unready pharmacists and what could be considered to increase the percentage of ready and qualified community pharmacists.

## CONFLICT OF INTEREST

The authors declare no conflict of interest.

## Data Availability

The data that support the findings of this study are available on request from the corresponding author, Saja A. Alnahar.
